# Genomic analysis of an Ecuadorian individual carrying an SCN5A rare variant

**DOI:** 10.1186/s12872-024-04049-w

**Published:** 2024-07-27

**Authors:** Santiago Cadena-Ullauri, Patricia Guevara-Ramírez, Viviana A. Ruiz-Pozo, Rafael Tamayo-Trujillo, Elius Paz-Cruz, Daniel Simancas-Racines, Rita Ibarra-Castillo, José Luis Laso-Bayas, Ana Karina Zambrano

**Affiliations:** 1 Centro de Investigación Genética y Genómica, Facultad de Ciencias de la Salud Eugenio Espejo, Universidad UTE, Quito, Ecuador; 2 Centro de Investigación en Salud Pública y Epidemiología Clínica (CISPEC), Facultad de Ciencias de la Salud Eugenio Espejo, Universidad UTE, Quito, Ecuador; 3 Clinical Cardiac electrophysiologist, Quito, Ecuador

**Keywords:** Genomic, Ancestry, *SCN5A*, Channelopathies, Ecuador

## Abstract

**Background:**

Ion channels, vital transmembrane protein complexes, regulate ion movement within cells. Germline variants in channel-encoding genes lead to channelopathies. The sodium channels in cardiac cells exhibit a structure of an alpha subunit and one to two beta subunits. The alpha subunit, encoded by the *SCN5A* gene, comprises four domains.

**Case presentation:**

A fifteen-year-old Ecuadorian female with atrial flutter and abnormal sinus rhythm with no familial history of cardiovascular disease underwent NGS with the TruSight Cardio kit (Illumina). A likely pathogenic *SCN5A* gene variant (NM_188056.2:c.2677 C > Tp. Arg893Cys) was identified, associated with arrhythmias, long QT, atrial fibrillation, and Brugada syndrome. Ancestral analysis revealed a predominant European component (43.9%), followed by Native American (35.7%) and African (20.4%) components.

**Conclusions:**

The participant presents atrial flutter and conduction disorders, despite lacking typical cardiovascular risk factors. The proband carries a *SCN5A* variant that has not been previously reported in Latin America and may be associated to her phenotype. The documented arginine-to-cysteine substitution at position 893 in the protein is crucial for various cellular functions. The subject’s mixed genetic composition highlights potential genetic contributors to atrial flutter, emphasizing the need for comprehensive genetic studies, particularly in mixed populations like Ecuadorians. This case underscores the importance of genetic analysis for personalized treatment and the significance of studying diverse genetic backgrounds in understanding cardiovascular diseases.

## Background

Ion channels are transmembrane protein complexes that facilitate the movement of ions between the interior and exterior of cells or cell organelles. They play an important role in maintaining cellular homeostasis, signal transduction, neurotransmitter release, muscle contraction, cardiac pacemaking, hormone secretion, growth, and apoptosis [1–3].

Autoimmune damage or germline variants in genes encoding sodium, calcium, potassium, and TRP channels can lead to dysfunctions in these protein complexes. Malfunction of ion channels, which are found in the membranes of all cells and many cell organelles, could lead to a variety of genetic, autoimmune, or inflammatory disorders known as channelopathies [1–3].

Ion channels are fundamental in the establishment of the membrane potential by facilitating the flow of ions across membranes, generating electrical currents [1, 3, 4]. In cardiac tissue, action potentials rely on the careful balance of different ionic currents. Disruptions to this equilibrium can lead to various cardiac arrhythmic disorders [5]. Cardiac channelopathies account for approximately 50% of sudden arrhythmic death syndrome cases and at least 1% of sudden infant death syndrome cases [3].

The action potential, which starts cardiac muscle contraction and keeps the heartbeat regular, is correlated with the opening and closing of sodium channels in cardiac cells [1, 3, 5]. Sodium channels remain closed when the cell is at rest, with the interior being negatively charged relative to the exterior. Upon reaching an electrical stimulation threshold, sodium channels open, allowing sodium ions to enter the cell and causing membrane depolarization. The rapid influx of sodium ions drives the rising phase of the action potential. Subsequently, sodium channels close, facilitating repolarization and returning the cell to its resting state [1, 6].

The sodium channels in cardiac cells exhibit a complex structure, consisting of an alpha subunit, responsible for forming the sodium pore (NaV1.5) and one to two beta subunits. The alpha subunit, encoded by the *SCN5A* gene, comprises four domains and each domain consists of six transmembrane helices. The voltage sensor is composed of the first four helices, while the final two helices and a P-loop form the pore. An intracellular linker connecting domains 3 and 4 links the voltage sensor to the pore [6–8].

NaV1.5 plays a critical role in the initiation and propagation of the action potential in cardiac muscle cells. Variants in the encoding gene are associated with various cardiac disorders, such as long QT syndrome 3, Brugada syndrome (BrS) 1, atrial flutter, familial atrial fibrillation 10, familial ventricular fibrillation 1, sick sinus syndrome (SSS) 1, heart block, dilated cardiomyopathy 1E, among others [9–12].

The activity of sodium channels in heart cells can be altered by variants in the *SCN5A* gene. Gain-of-function variants, such as those associated with long QT syndrome 3, result in a persistent sodium current during repolarization, prolonging the action potential and QT interval on the electrocardiogram (ECG). On the other hand, loss-of-function variants lead to a reduction of sodium current, affecting cell depolarization and impairing the generation and transmission of electrical impulses, influencing the timing and coordination of cardiac contractions (e.g., BrS 1 and atrial flutter). In addition, both types of variants may contribute to the development of dilated cardiomyopathy, an arrhythmogenic disease with gross structural defects of the left ventricle [10, 12].

The clinical phenotypes associated with *SCN5A* gene variants-related channelopathies exhibit different characteristics. Atrial flutter is identified by the rapid and regular beating of the cardiac atrium, while ventricular or atrial fibrillation is marked by rapid and irregular beating of the ventricle or atrium of the heart. Irregular heartbeats, pauses, or slow heart rhythms are indicative of SSS. There may also be an abnormality in ventricular repolarization, typical of LQTS, which increases the risk of sudden death and the development of polymorphic ventricular tachycardia (torsades de pointes) [13]. Conversely, ST-segment elevations in the precordial leads of the ECG are indicative of BrS [14–19]. Despite their specific types, all these disorders raise concerns about the potential development of life-threatening arrhythmias that could result in sudden cardiac death.

This case report is the first one that describes an Ecuadorian woman with atrial flutter and a mainly European ancestral component. Moreover, the identified variants have not been reported in any Latin American country. The study used Next-Generation-Sequencing (NGS) to uncover a rare variant in the *SCN5A* gene, suggesting a potential correlation with the observed clinical phenotype. This finding contributes to the understanding of the genetic factors of cardiovascular diseases in populations with mixed genetic backgrounds.

## Case presentation

This case report study was conducted following the approval of the Ethics Committee of Universidad UTE. The participant provided signed informed assent, while the legal guardian gave informed consent for NGS, ancestral analysis, and inclusion of the proband’s clinical data in publications.

The subject is a fifteen-year-old Ecuadorian female diagnosed with atrial flutter and abnormal sinus rhythm. Her clinical history revealed an unspecified heart whistling sound at birth, which was better characterized as a heart murmur at five months old. Until the age of ten, normal cardiac functionality was reported. However, at thirteen years old, an arrhythmic cardiac event was detected during an ECG test. Furthermore, the subject and her immediate family mentioned no history of cardiovascular disease, including arrhythmias and sudden death. However, two great-grandparents had an unspecified heart condition.

The EKG revealed an atrial flutter rhythm with a heart rate of 120 beats per minute, a QRS axis of -90 degrees, and an incomplete right bundle branch block (IRBBB) with left anterior superior hemiblock. The QT interval was within normal ranges. Propranolol was prescribed, and a transesophageal echocardiogram was performed. The echocardiogram results showed a structurally and functionally normal heart, with no atrial or cava thrombi, mild tricuspid insufficiency, and mild left ventricular systolic dysfunction. Atrial flutter was diagnosed, leading to the suspension of Propranolol for subsequent catheter ablation.

Simultaneously, a genomic test revealed a likely pathogenic variant in the *SCNA5* gene. After catheter ablation, the proband recovered satisfactorily, and a normal sinus rhythm was established. Moreover, no pharmacological treatment was prescribed, and no exercise limitations or lifestyle changes were recommended.

**Fig. 1:**
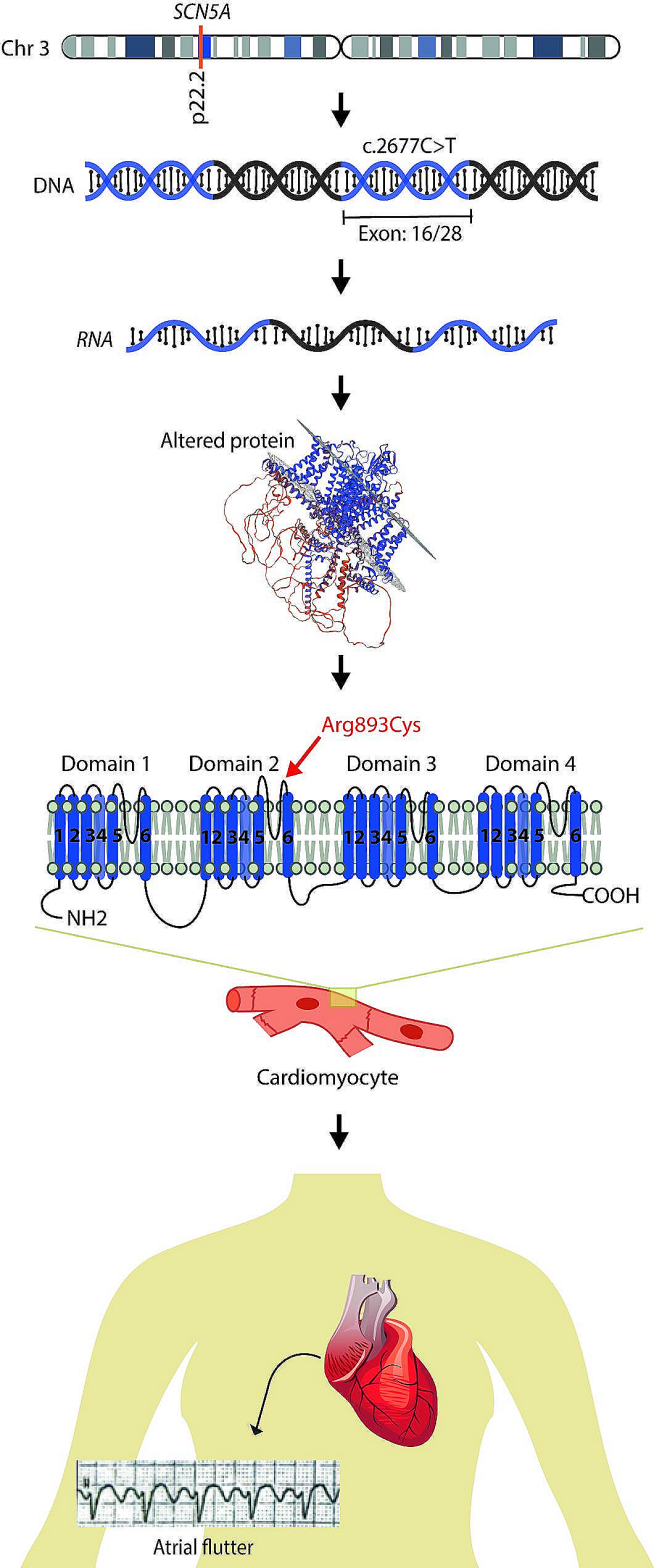
Timeline of participant’s clinical episodes and treatment strategies

However, 6 months after the ablation, the patient shows electrocardiographic evidence of sinus node disease, manifested by sinus pauses and escape rhythms, also a first-degree AV Block. No QT interval deviations were registered. These findings are critical for future therapeutic planning, suggesting the potential need for further interventions such as pacemaker implantation to stabilize the heart rhythm and prevent adverse events. Therefore, it is crucial that the patient undergo a distinct test to assess the presence of BrS and determine appropriate treatment options (Fig. 1). Even though the sinus node disease and first-degree AV block were detected after ablation, no pharmacological or physical restrictions were recommended. However, periodical EKG controls are performed to detect cardiac rhythm alterations, which could justify the implementation of more specific interventions.

### Molecular assays

A peripheral blood sample in an EDTA anticoagulant tube was collected. Subsequently, DNA extraction was performed with the PureLink Genomic DNA Mini Kit (Life Technologies) according to the manufacturer’s guidelines [20]. DNA integrity was assessed using gel electrophoresis, while DNA quantity and quality were determined using NanoDrop™ 2000 spectrophotometer and Qubit 4 fluorometer (Thermo Scientific™) quantifications. The DNA was then diluted to a concentration of 5ng/µL for further processing in the NGS protocol.

For the genomic assay, we used the TruSight Cardio kit (Illumina) that covers 174 genes associated with 17 Inherited Cardiac Conditions following the manufacturer’s guidelines [21] in the MiSeq System (Illumina) equipment.

The ancestral proportions analysis was performed with 46 ancestral informative INDEL markers in one multiplex reaction as previously described [22] in a 3500 Genetic Analyzer (Thermo Fisher Scientific).

### Data analysis

The results for the ancestry proportion analysis were obtained using the Data Collection v.3.3 software, and the electropherograms were analyzed using the Gene Mapper v.5 software. Ancestry inference for the proband was conducted using STRUCTURE v.2.3.4, where the proband’s allele frequency was compared with the frequencies observed in Africans, Native Americans, and Europeans.

Genomic data analyses were performed using Dragen Enrichment v.4.2.4, aligning with the reference genome (GRCh38), to identify genetic variants associated with the proband’s phenotype. The identified genetic variants were further analyzed using Variant Interpreter Software (Illumina), which classified them into variants of uncertain significance (VUS), likely pathogenic, or pathogenic.

### Diagnostic assessment

#### Genomic results and ancestry composition

**Fig. 2:**
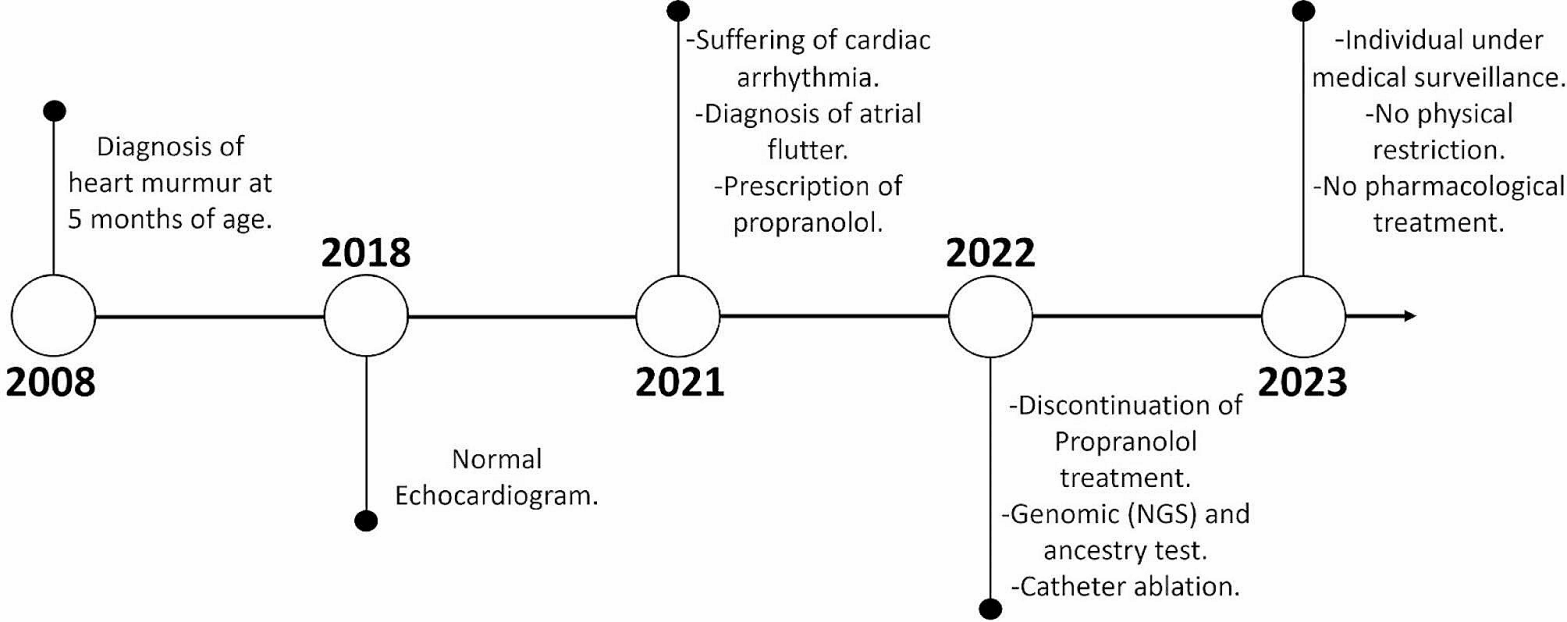
*SCN5A* rare variant associated with atrial flutter on an Ecuadorian woman. The image describes the molecular aspects of the described *SCN5A* variant

In the NGS genomic screening performed on the individual using the TSC Sequencing panel, 95.35% of target regions had a coverage ≥ 50 X. The analysis identified a likely pathogenic variant in the *SCN5A* gene in exon 16/28 (NM_188056.2:c.2677 C > Tp. Arg893Cys). Variants in the *SCN5A* gene are associated with arrhythmias, long QT, atrial fibrillation, BrS, and flutter (Fig. 2).

**Fig. 3:**
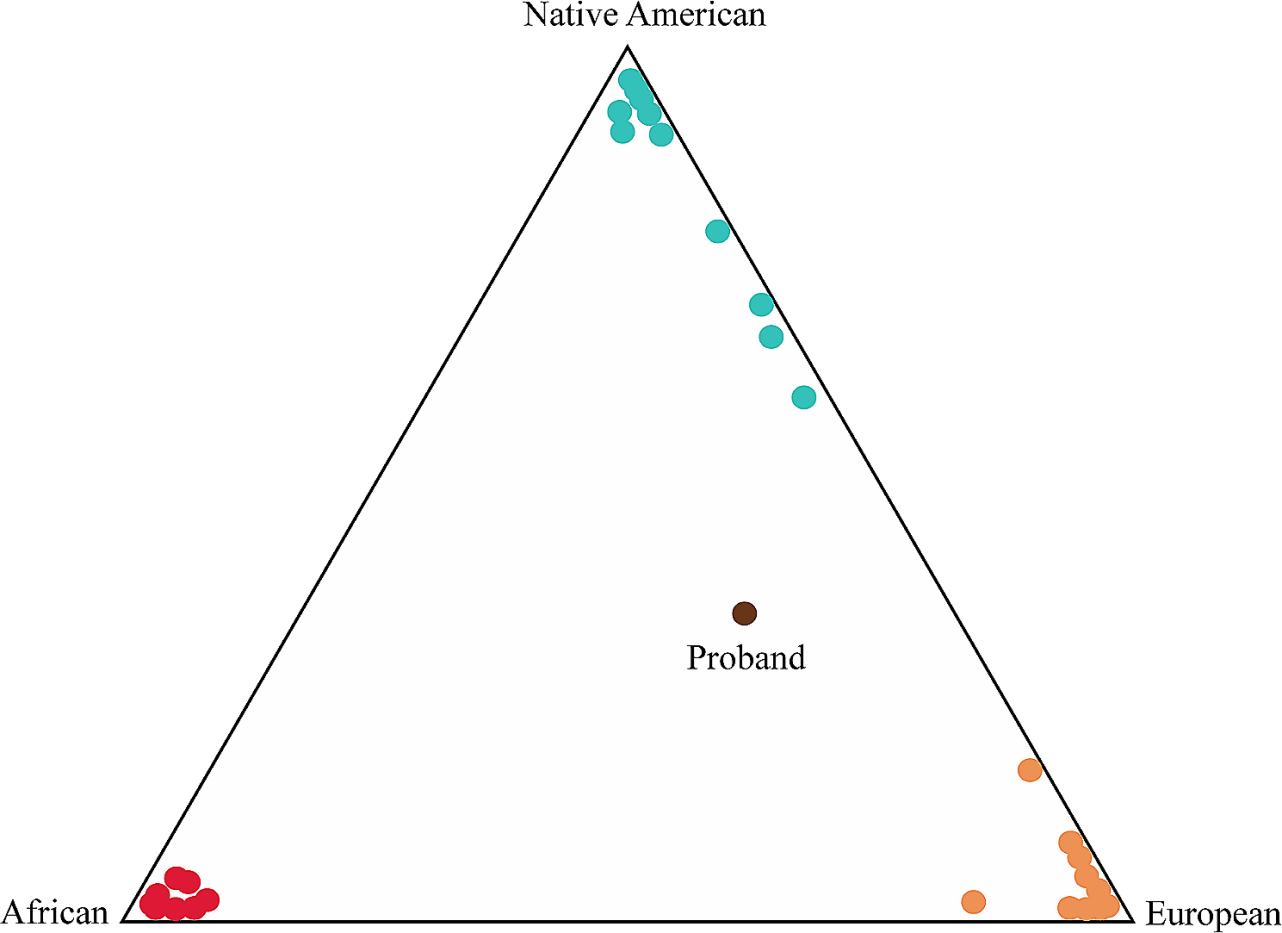
Ancestry analysis. Proband’s ancestry composition. The proband is shown in brown, African ancestry in red, Native American in orange, and European in light blue

Furthermore, the results of the proband’s ancestral composition indicated a higher European component (43.9%), followed by the Native American component (35.7%) and African component (20.4%) (Fig. 3).

## Discussion

The gene *SCN5A* encodes the alpha subunit of the pore-forming ion cardiac sodium channel (NaV1.5). This sodium channel is predominantly expressed in the cell membrane of cardiomyocytes, facilitating the influx of Na + ions from the extracellular environment into the cell [12, 23]. Consequently, NaV1.5 plays a pivotal role in regulating the depolarization of Purkinje, ventricular, and atrial myocytes, as well as in maintaining the conduction of electrical impulses within the atria and ventricles [12].

Variants in the *SCN5A* gene have been linked to various cardiovascular phenotypes and diseases, including long QT syndrome, SSS, dilated cardiomyopathy, and atrial flutter, among others [24, 25]. Atrial flutter, characterized by an elevated atrial rate accompanied by variable or fixed ventricular rate, ranks among the most common arrhythmias [26]. Established risk factors associated with atrial flutter encompass male sex, advancing age, alcohol consumption, hypertension, and obesity. However, the participant described in this case report lacks these risk factors; she is a young female with normal blood pressure, weight, and no alcohol intake. Conversely, emerging risk factors for atrial flutter include familial and genetic components, as observed in this case with two great-grandparents with unspecified heart conditions [27].

In the present case, by performing NGS a possibly pathogenic variant in the *SCN5A* gene (NM_188056.2, c.2677 C > T, p.Arg893Cys) was identified (Table 1). This alteration at codon 893 of the SCN5A protein substitutes the basic and polar arginine with the neutral and slightly polar cysteine. The variant frequency is < 0.001% in the whole gnomAD population, but specifically in African Americans (gnomAD 0.0001%) and Africans (gnomAD 0.0001%). Polyphen2 predicted the variant as likely pathogenic (score 0.96). The missense variant is dispersed in segments S5 to S6 of the DII domain, which includes the pore and selectivity filter of the sodium channel. This variant affects the amino acid residue p.Arg893 in SCN5A (ClinVar Variation ID: 67,748), potentially impacting the proper function of the sodium channel pore [24, 28].

**Table 1:** Genetic variant detected in the patient associated with arrhythmia

Gene	Variant	Consequence	dbSNP	Association SIFT/PolyPhen	ACMG
SCN5A	SNV chr3:38585801	Missense NM_198056.2 c.2677 C > T p.(Arg893Cys)	rs199473171	Deleterious / Probably Damaging	Likeky Pathogenic PM1 PM2 PP3 PP4

This constitutes the first report of this variant in an Ecuadorian subject diagnosed with atrial flutter. Furthermore, the variant has not been previously documented in the Latin American population. The variant described in this manuscript has not been associated with atrial flutter, but, according to ClinVar, has primarily been correlated with BrS, despite the *SCN5A* gene being related to other cardiovascular diseases [24, 29–32].

Brugada syndrome, characterized by an autosomal dominant inheritance, presents ST-segment elevation and an augmented risk of sudden cardiac death. Approximately, 30% of the BrS cases are attributed to variants in the *SCN5A* gene [12, 33]. Notably, the literature suggests that atrial flutter may manifest as the initial symptom in BrS 1 [33–35]. Furthermore, atrial flutter has been linked to an increased risk of developing malignant arrhythmic events in BrS [35]. Furthermore, it is noteworthy that the prevalence of atrial flutter in individuals with BrS is 20% in those under 40 years of age. This observation raises the intriguing possibility that our clinical case subject may potentially develop BrS in the future [36]. Consequently, it is essential that the patient undergoes the Ajmaline provocation test in the near future to determine if BrS develops. This can help prevent possible complications like the risk of sudden death and ensure appropriate treatment [37].

Notably, Aizawa et al. previously reported this variant in a member of a Japanese family with a familial history of BrS; this individual, who was the sole participant undergoing genetic analysis, presented the NM_188056.2:c.2677 C > T, p.Arg893Cys variant in the *SCN5A* gene. Intriguingly, this individual received a diagnosis of SSS at the age of 40, and following electrical cardioversion, atrial flutter was observed [38]. Although there is insufficient evidence between the association of BrS and SSS.

Hayashi et al. also described a patient with both diagnostics, BrS and SSS, and reported that BrS may be an arrhythmogenic disorder involving not only the His-Purkinje system and the right ventricle but also the sinus node and the atrium, derived from variants in ion channels such as the sodium channel gene *SCN5A*. In addition, arrhythmias, such as atrial flutter in BrS, are usually seen with *SCN5A* variants [39, 40]. Unfortunately, the information regarding this patient is limited. However, by analyzing the available data, we identified that the patient in this case report, shares an IRBBB, similar to the patient studied by Hayashi. Moreover, other studies have associated the presence of an *SCN5A* variant and sick sinus dysfunction (SSD) and SSS [41–45].

In this case report, a substitution from arginine to cysteine change is documented at position 893 within the protein. Arginine, a positively charged amino acid, is primarily located on protein surfaces. This amino acid has a guanidinium group and a particular type of aromatic system in its structure that allows it to form five hydrogen bonds and salt bridges with other compounds including water. Notably, arginine plays a crucial role in processes such as proper protein function, nucleic acid recognition, protein-ligand binding, and protein-protein oligomerization [46, 47].

Conversely, cysteine, characterized by a thiol group instead of guanidinium, is mainly associated with disulfide bond formation. While cysteine can form hydrogen bonds similar to arginine, its thiol group allows for the formation of three hydrogen bonds, in contrast to arginine’s five [47, 48].

Hence, arginine plays a key role in various physiological processes, particularly influencing the proper function of proteins. In this context, its specific location and interactions are pivotal for the optimal functioning of the cardiac sodium channel by contributing to the formation of hydrogen bonds and salt bridges, essential for the channel’s stability and conductance of sodium ions [46, 47]. Moreover, variants, like the one described in this case report, have the potential to disrupt the protein’s structure, function, and interactions, leading to abnormal cardiac electrophysiology and arrhythmias [46, 47].

Furthermore, the genetic ancestry of the individual revealed proportions of 20.4% African, 43.9% European, and 35.7% Native American, indicating a significant deviation from the previously documented ancestral composition of the general Ecuadorian population [49]. The observed elevated African genetic component observed in this individual is particularly noteworthy.

The increased African genetic proportion in our study subject is consistent with findings from genetic studies involving the African-American population. A study by Cheng et al. (2011) reported *SCN5A* variants in African-American individuals, suggesting convergence and their association with arrhythmic sudden death [50].

Furthermore, the identified variant in the individual under analysis is clinically significant for BrS, a condition predominantly observed in Asian populations [51, 52]. The prevalence reported in Asians is approximately five times higher than in Caucasians and 32 times higher than in Hispanics [53]. The widely accepted hypothesis attributing the elevated prevalence of BrS in Asia implicates ethnic-specific polymorphisms that may modulate the activity of a crucial protein, thereby contributing to disease manifestation [52, 54].

In summary, the mixed genetic composition observed in the individual under analysis offers valuable insights into potential genetic contributors to atrial flutter. The association of ethnic-specific genetic markers emphasizes the need for comprehensive genetic studies, especially in mixed populations such as Ecuadorians.

Hence, the integration of ancestry studies with genomic analyses, employing NGS, holds the potential to comprehensively characterize the Ecuadorian population. This approach can aid in understanding the differences in the incidence of cardiovascular diseases, their impact by age or gender, and responses to treatments compared to other populations.

NGS stands as a valuable tool in medical settings, providing insightful information on diagnosis, prognosis, and aiding in the development of targeted treatments tailored to individual genetic profiles. The American Heart Association actively promotes research in cardiovascular genomics and genetics to deepen our understanding of the impact and role that these have on heart-related diseases [55, 56].

Furthermore, NGS offers various advantages over similar methods like Sanger sequencing. For instance, NGS provides high-yield results, enabling the identification of novel, previously undescribed variants associated with cardiovascular diseases [57, 58]. However, NGS also has limitations; for example, the need for bioinformatic tools and that the results may not always be conclusive. Given the complexity of the human genome, many variants may be categorized as variants of uncertain significance, necessitating further studies to identify their impact on protein structure and function [57, 58].

The diagnosis of genetic diseases that follow a Mendelian inheritance pattern can be challenging, especially in low-income countries. For instance, in Ecuador there has been an increase of infant mortality [59], which could be attributed to sub-diagnosis of genetic diseases. Moreover, by implementing the use of Next Generation Sequencing (NGS), single variants associated with the individual clinical phenotype can be identified [60]. This approach has the potential to significantly improve the diagnosis of genetic diseases and guide a personalized treatment, reducing the cost of healthcare and the time of hospitalization [61]. Hence, the identification of the aforementioned *SCN5A* variant, through NGS in this proband, has provided a deeper understanding of the clinical phenotype, risks associated and the best treatment route, leading to an improvement in the patient’s general condition.

## Conclusion

In conclusion, the present case report highlights the significant role of the *SCN5A* gene and its association with cardiovascular diseases. The subject of this study has been diagnosed with atrial flutter, and by performing NGS, a likely pathogenic variant was identified. Furthermore, the participant exhibits a higher proportion of African ancestry, which may suggest that ethnic-specific genetic variants could contribute to disease manifestation. This case emphasizes the need for comprehensive genetic studies in diverse populations like the Ecuadorian. Moreover, NGS can be a valuable tool in understanding the complexity and impact of genetic variants on human health.

### Patient’s perspective

The legal guardians of the study expressed gratitude to the researchers for helping them to understand the disease and its genetic aspects.

## Data Availability

All data generated or analyzed during this study are included in this published article. For more information, please contact the corresponding author AKZ (anazambrano17@hotmail.com).

## Abbreviations

NGS: Next Generation Sequencing
ECG: electrocardiogram
IRBBB: incomplete right bundle branch block
VUS: variants of uncertain significance
SSS: sick sinus syndrome
